# Gene Regulation of Iron-Deficiency Responses Is Associated with Carbon Monoxide and Heme Oxydase 1 in *Chlamydomonas reinhardtii*

**DOI:** 10.1371/journal.pone.0053835

**Published:** 2013-01-22

**Authors:** Zhang Liping, Shao Hongbo, Long Xiaohua, Liu Zhaopu

**Affiliations:** 1 Jiangsu Key Lab of Marine Biology, College of Resources and Environmental Sciences, Nanjing Agricultural University, Nanjing, China; 2 Key Laboratory of Coastal Biology & Bioresources Utilization, Yantai Institute of Costal Zone Research(YIC), Chinese Academy of Sciences (CAS), Yantai, China; 3 Institute of Life Sciences, Qingdao University of Science and Technology, Qingdao, China; University of South Florida College of Medicine, United States of America

## Abstract

Carbon monoxide (CO) as an endogenous gaseous molecule regulates a variety of biological processes in animals. However, CO regulating nutrient stress responses in green alga is largely unknown. On the other hand, heme oxydase (HO1 as a rate-limiting enzyme of the first step for heme degration and to catalyze heme into biliverdin (BV), which is concomitant with releasing of CO and ferrous ions, probably participates in the process of CO-regulating response to nutrient stress in green alga. In this paper, we described an observation that CO could regulate iron-homeostasis in iron-starving *Chlamydomonas reinhardtii*. Exogenous CO at 8 µM was able to prevent the iron deficient-inducing chlorosis and improve chlorophyll accumulation. Expression pattern of FOX1, FTR1 and ferredoxin was up-regulated by CO exposure in iron-deficient mediam. treatment with external CO increasing iron accumulation in iron-deficient *C. reinhardtii*. Moreover, to get insights into the regulatory role of HO1, we constructed a transgenic alga overexpressing HO1 and HO1 knock-out mutants. The results show that there was no significant influence on chlorosis with HO1 overexpression of *C. reinhardtii* under iron-deficiency and the chlorophyll accumulation, and gene expression associated with iron deficiency of mutant were greatly improved. Otherwise, those results from HO1 knock-out mutants were opposite to HO1 overexpression mutants. Finally, CO exposure induced NO accumulation in cells. However, such an action could be blocked by NO scavenger cPTIO. These results indicate that CO/HO1 may play an important role in improving green algae adaptation to iron deficiency or cross-talking with NO under the iron deficiency.

## Introduction

Iron (Fe) as an essential element is required for various cellular and physiological processes from respiration to photosynthesis. However, abundant iron is often unavailable for crops due to its low solubility of oxidized form (Fe^3+^) in farmland. On the opposite, anaerobic conditions in acidic soils may lead to cellular iron overload, triggering toxicity to plants [1]. Thus, the iron levels in plant cells must be strictly regulated. Iron abundance in plants is primarily regulated by uptake, translocation and recycling. In *Arabidopsis* (strategy I plant species), iron uptake is controlled by at least three steps, including acidification of the rhizosphere by an H+-AT-Pase, reduction of Fe (III) to Fe (II) by ferric chelate reductases, and uptake of Fe (II) by transporters, while in *Chlamydomonas reinhardtii*, mobilization of iron from extracellular domain to intracellular fraction is involved in reduction of Fe by ferrireductases at the cell surface, and transported by iron transporters, like FTR1 [2].

For high plants and green algae, insufficient iron uptake occurs in most types of soils and medium conditions. This may lead to chlorosis, a typical iron-deficient symptom. Under the iron starvation a range of genes related to the iron acquisition are induced [3]–[7]. Expressions of AtFRO2, LeFRO1 and PsFRO1 by coding ferric-chelate reductase were up-regulated by iron deficiency in *Arabidopsis*, tomato and pea [4], [8]–[10]. Moreover, the expression of Ferrireductases, ferredoxin and Ftr1 were also up-regulated by iron deficiency in *C. reinhardtii*. Additionally, iron reduction at the cell surface is a key step in mobilizing iron for uptake. *Chlamydomonas* Ferrireductases gene coding an iron transporter was found to be a major route for iron entering the cell [2]. One of the key players in the storage process is ferritin. The expression of ferritin is tightly regulated according to the cellular iron status. With regard to the iron acquisition gene under iron deficiency, an essential gene named Fox1 has been recently characterized; which shows highest sequence similarity to the mammalian ceruloplasmins and hephaestins and founction in iron assimilation.

Although iron uptake and transportation are controlled by many components which have been recently characterized at the molecular level [11], little is known about *Chlamydomonas reinhardtii* signal network of the iron deficiency. Nitric oxide (NO) as a crucial signaling molecule participates kinds of interactions in many biotic and abiotic stresses in plant development procedures, including pollen tube growth [12]. Recent studies have shown that NO regulates iron homeostasis via modulating ferritin synthesis in animals; also, some other evidences have been provided and proved that carbon monoxide (CO) also participates in the process [13]. Several studies demonstrated that NO may regulate iron metabolism in plants [14], [15] and green alga [16], however, no report is available about CO, which regulates plant and alga iron homeostasis. Recently, CO has been highly appreciated for its versatile properties as a signalling mediator in animals [17]. Meanwhile, CO is shown to be endogenously generated and participate in regulating plant growth and development in plants [18], [19]. Generation of intracellular CO is closely linked to heme oxygenase (HO, EC 1.14.99.3), which catalyze the degradation of heme to produce CO, free iron and biliverdin [20]. The iron derived from heme is thought to be the endogenous source of Fe incorporated by proteins. Biliverdin, the final HO product, is reduced to bilirubin by bilirubin reductase [21]. Genetic analysis has indicated that bilirubin takes part in synthesis of the bilin chromophores for assembling photochemically-active components [22]. Up to now, there have been two HO members (HO-1 and HO-2) isolated from *Chlamydomans teinhardtii* [23]. *Chlamydomonas* HO-1 (HMOX1) has been well characterized, and its expression is induced by numerous stimuli. The other gene (HMOX2) with different patterns of expression displays different sequence similarity to HMOX1. In our study, we identified the role of CO in regulating the adaptive response to iron deficiency in wild-type *Chlamydomonas* and transgenic one. Genetic and transcriptional analysis supported the role of CO/HO1 in modulating phenotype and the expression of genes controlling iron homeostasis in *Chlamydomonas*. Finally, pharmacological and molecular evidence further verified the cross-talk between CO and nitric oxide (NO).

## Results

### CO/HO1 prevents chlorosis in iron-deficient algae

**Figure 1:**
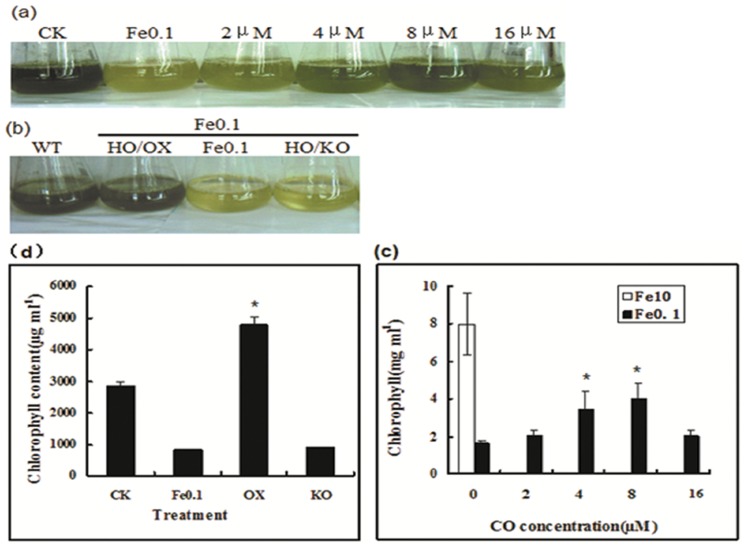
Carbon monoxide/heme oxygenase regulation of phenotype (a), (b) and chlorophyll levels (c), (d) of *C. reinhardtii* under the iron-deficiency. The cells were cultivated under the condition of sufficient Fe (10 µM) and deficient Fe (0.1 µM) with or without CO (8 µM) for 7 days. After that, the photographs were taken and the chlorophyll was quantified. Vertical bars represent standard deviation of the mean (n = 3). Asterisk indicates that mean values are significantly different between the treatments of Fe0.1+CO or HO/OX, HO/KO and Fe0.1 (control) (P<0.05).

**Figure 2:**
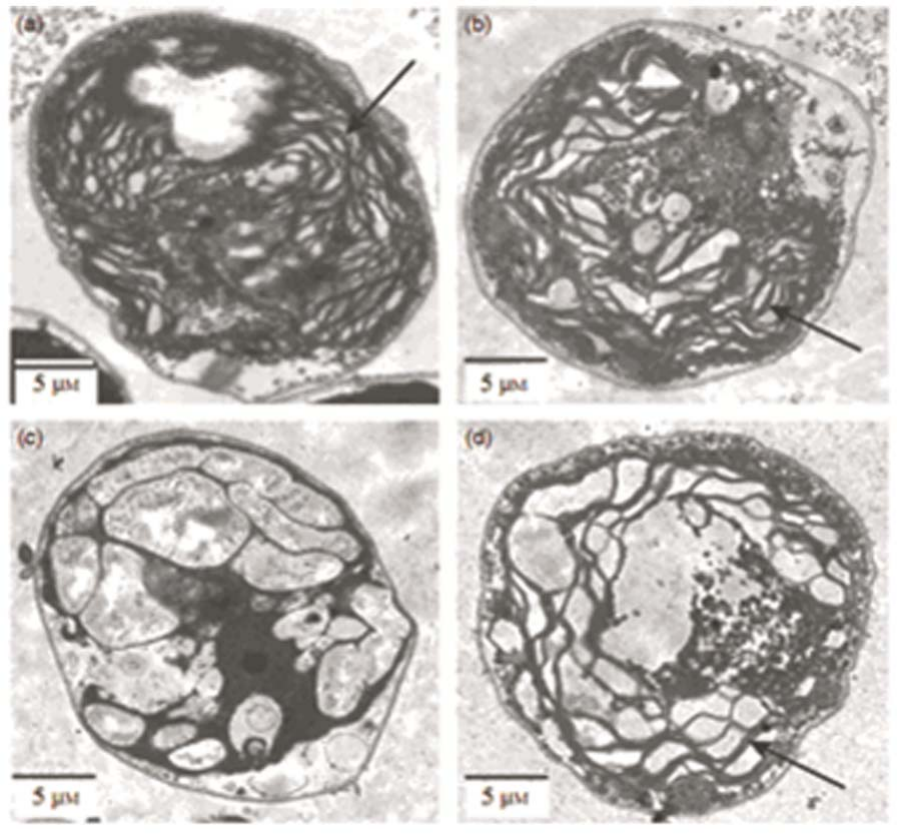
Carbon monoxide improves the internal structure of *C. reinhardtii* chloroplasts under iron deficiency. Cells were cultured under the condition of sufficient Fe (10 µM) (a and b) and deficient Fe (0.1 µM) (c and d) for 7 days. a: cells supplied with 10 µM Fe; b: cells with 10 µM Fe + 8 µM CO; c: cells with 0.1 µM Fe; and d: cells with 0.1 µM Fe + 8 µM CO. After treatment, the cells were visualized under TEM and photographed. The micrographs were zoomed in to show the thylakoid (as indicated with arrows) inside the chloroplasts.

**Figure 3:**
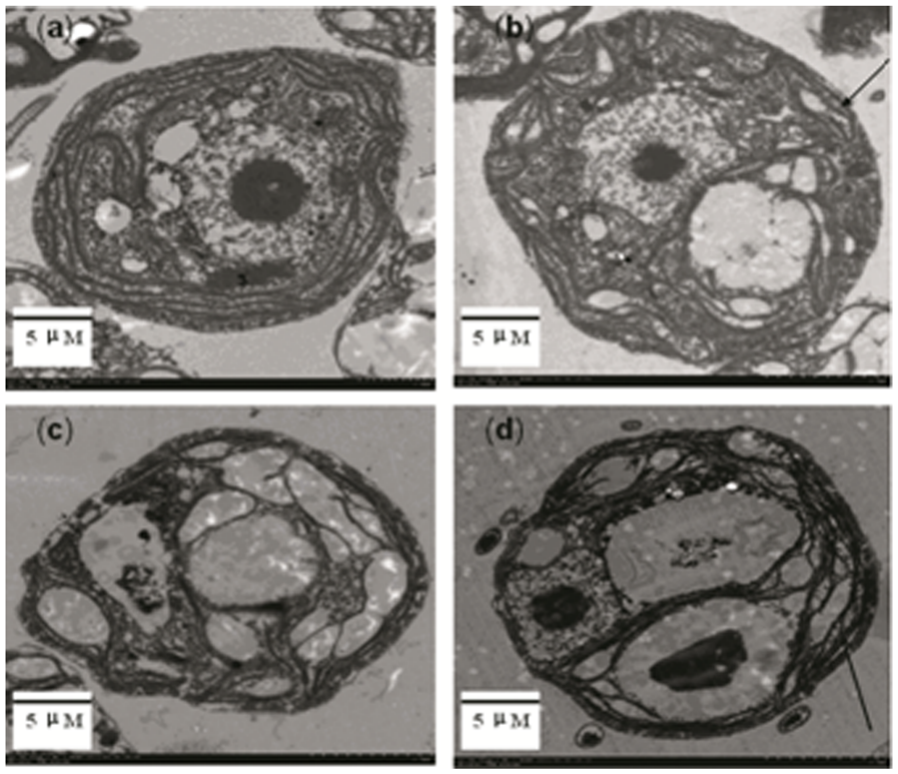
Heme oxygenase improves the internal structure of transgenic *C. reinhardtii* chloroplasts under iron deficiency. Cells were cultured under the condition of sufficient Fe (10 µM) (a) and deficient Fe (0.1 µM)(b, c and d) for 7 days. a: wild-type cells supplied with 10 µM Fe; b: HO/OX mutant cells with 0.1 µM Fe; c: wild-type cells with 0.1 µM Fe; and d: HO/KO mutant cells with 0.1 µM Fe. After treatment, the cells were visualized under TEM and photographed. The micrographs were zoomed in to show the thylakoid (as indicated with arrows) inside the chloroplasts.

Just because chlorosis is a remarkable symptom of iron deficiency, firstly, we tested whether exogenous CO could improve cell greening in iron-deficient algae. The green alga *C. reinhardtii* is a model of unicellular eukaryotic cells and widely used to study metal homeostasis, particularly iron metabolism in chloroplasts [24], [25]. Iron deficiency in medium induced bleaching and reduced the cell growth. However, treatment with CO at 2–8 µM greatly improved the growth rate and chlorophyll levels (Figure 1a). To learn more about the possible role of CO in improving chlorophyll accumulation under iron deficiency, we constructed transgenic *Chlamydomonas* named Chlamy HO/OX (overexpression) and the other HO/KO (knocking out) as comparison. We found that HO/OX completely performed green under iron deficiency, while HO/KO showed more serious chlorosis compared to wild-type (Figure 1b). This result suggests that CO/HO1 also regulates cellular iron status in algae. The CO-improved cell greening was estimated by measuring chlorophyll content. A 3.64-fold increase was achieved in the wild-type cells fed with CO (Figure 1c) and 2.13-fold increase was achieved in HO/OX mutant cells (Figure 1d). A concentration-dependent change was found in chlorophyll content. To understand further possible function of CO/HO1 in preventing chlorosis of iron-deficienct *Chlamydomonas*, we performed electron microscopy analysis of chloroplast ultrastructure in *C. reinhardtii* cells. Our analysis with transmission electron microscopy (TEM) indicated that the majority of thylakoids inside iron-starved chloroplasts was disrupted (Figure 2c, Figure 3c), which was also the same to HO/KO mutant cells (Figure 3d). In contrast, most of thylakoids in chloroplasts with CO (Figure 2d) and HO/OX mutant cells (Figure 3b) remained in integrity. Furthermore, the iron-deficient cells and HO/KO mutant cells had chloroplasts full of starch granules or plastoglobuli, whereas in CO-exposed cells and HO/OX mutant cells there were relatively fewer or smaller granules. These results indicate that CO/HO1 improved the ultrastructure of chloroplasts and consequently improved chlorophyll accumulation.

### CO regulates expression of genes related to iron acquisition

**Figure 4:**
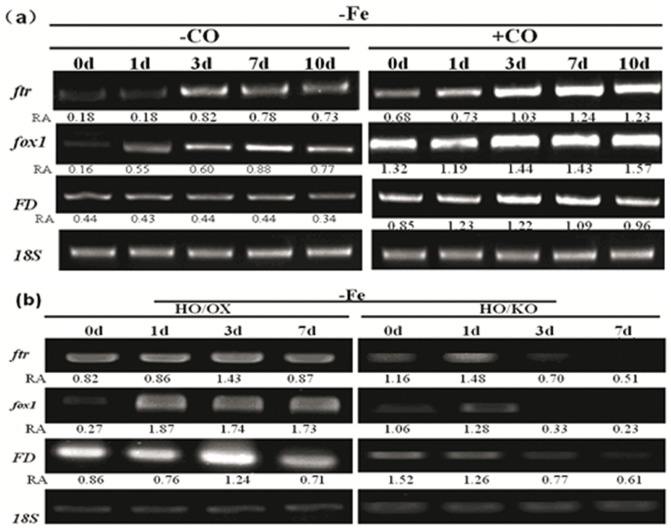
Carbon monoxide/heme oxygenase regulates Chlamydomonas FOX1, FTR1 and FD transcript levels in Fe-deficient cells. Cells were grown in iron-sufficient (Fe10) nutrient solution for 5 days, and then transferred to iron-limited (Fe0.1) media with or without 8 µM CO for 7 or 10 days. 18S was used for cDNA normalization. The number below the band indicates the relative abundance (RA) of the genes with respect to the loading control 18S.

Previous studies have demonstrated that Fe-deficiency in *Chlamydomonas* induces several major genes for iron acquisition and recycling [26]. To get insights into the regulatory role of CO under Fe-deficiency, we analyzed the expression of *FOX1*, *FTR1* and *FD*. Expression of these three genes was induced 5 days after iron deficiency (Figure 4a). Concomitant treatment with 8 µM CO resulted in additionally increased gene transcripts. In *Chlamydomonas*, FOX1 codes a plasma membrane protein named multicopper oxidase and it is generally associated with a permease or transporter component for delivery of iron across [2]. One of its partners is FTR1, whose expression pattern is up-regulated coordinately with Fox1 during Fe-deficiency [26]. Our results revealed that fox1 and ftr1 transcripts accumulated in response to iron deficiency and displayed higher levels in cells exposed to 8 µM CO. FD, performed in parallel and encoding ferredoxin, probably the most abundant iron protein in soluble extracts of iron-replete *Chlamydomonas* cells. A similar expression pattern was observed for transgenic mutants (Figure 4b). Our analysis indicated that iron status could be regulated by CO exposure.

### CO regulates multi-copper ferroxidase1 activity and iron accumulation

**Figure 5:**
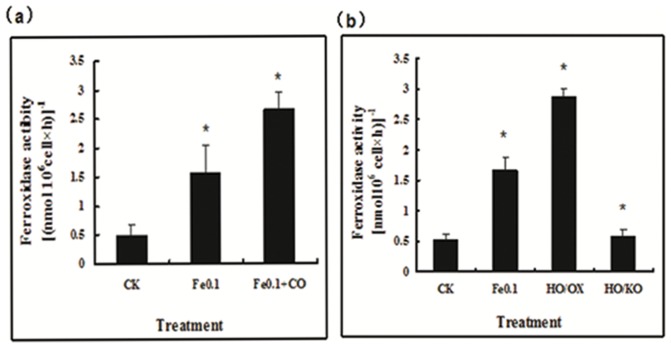
Effect of carbon monoxide (CO)/heme oxygenase (HO1) on the activity of multicopper ferroxidase1 in Chlamydomonas cells under iron deficiency. Cells were grown in the iron-sufficient (Fe10) nutrient solution for 5 days, and then transferred to iron-limited (Fe0.1) media with or without 8 µM CO for 7 days. Vertical bars represent standard deviation of the mean (n = 3). Asterisk indicates that mean values are significantly different between the treatments of Fe0.1+CO or HO/OX, HO/KO and Fe5 (control) (P<0.05).

**Figure 6:**
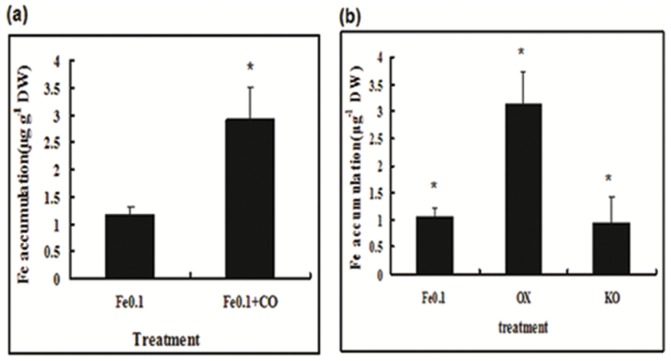
Effect of CO/HO1 on iron accumulation in *C. reinhardtii*. Cells were grown in the nutrient solution for 5 days, and then transferred to iron-limited (Fe0.1) media with or without 8 µM CO for 8 days. Thereafter, the total iron in whole cells was quantified. Vertical bars represent standard deviation of the mean (n = 3). Asterisk indicates that mean values are significantly different between the treatments of Fe0.1 + CO or HO/OX, HO/KO and Fe0.1 (control) (P<0.05).

In *C. reinhardtii*, Fe(III) is first reduced by ferric chelate reductases on the surface of cell membrane. On the contrast, the high affinity iron uptake is mediated by multi-copper ferroxidase that reoxidizes Fe(II) into Fe(III) and then Fe(III) is transported by iron permease into the cell, as it occurs in yeast and mammals [26], [27]. For this reason, multicopper ferroxidase1 plays an important role during high affinity iron uptake. Genetic analysis showed that Fe (III) chelate reductase was encoded by FOX1, which was expressed in PM. We assayed the multicopper ferroxidase1 activity in iron-deficient cells and observed that concomitant treatment with 8 µM CO significantly increased the activity of multicopper ferroxidase1 as compared to the treatment of iron-deficiency alone (Fe0.1, control) (Figure 5a), consistent with the data of *fox1* transcript (Figure 4a). Similarly, the multicopper ferroxidase1 activity in iron-deficient HO/OX mutant cells was much higher than that of the treatment of iron-deficiency alone (Fe0.1, control) and HO/KO mutant cells (Figure 5b). To determine whether iron actually accumulated in *Chlamydomanas* cells, we measured iron concentrations by using inductively coupled plasma spectrometry. The iron levels were significantly higher in the CO-treated cells than those of the control (iron-deficiency alone) (Figure 6a). Meanwhile, in the other work, the mutants showed parallel with wild-type (Figure 6b).

### Heme oxygenase 1 was up-regulated by iron deficiency

**Figure 7:**
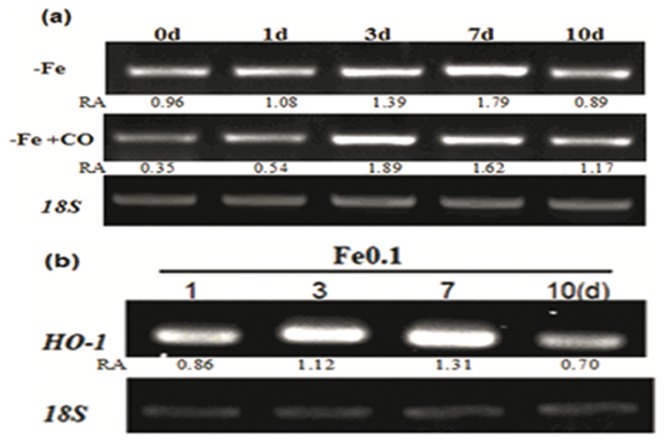
Iron deficiency up-regulates HO-1 expression of wild-type Chlamydomans (a) and transgenic mutant HO/OX (b). Cells were grown in iron-sufficient Fe (10 µM) nutrient solution for 5 days and then transferred to iron-limited Fe (0.1 µM) media for 0, 1, 3, 7 and 10 d. 18S was used for cDNA normalization. The number below the band indicates the relative abundance (RA) of the genes with respect to the loading control 18S.

In *Chlamydomonas*, heme oxygenase (HO) catalyzes the opening of the heme (iron protoporphyrin IX) ring at the alphamethene bridge to formbiliverdin IX, Fe^3+^, and carbon monoxide. The free Fe may be transferred and reused for chlorophyll synthesis in chloroplasts. In addition, HO-1-derived CO might mediate the iron availability in the same way as the external CO. Because HO-1 is transcriptionally regulated, expression level of HO-1 in Fe-deficient Chlamy cells was analyzed. In wild type, during the early stage of iron-deficiency (0–24 h), expression level of HO-1 was very low (Figure 7a). After a short time of induction, its expression pattern was up-regulated. In transgenic mutants of HO/OX, the expression of HO-1 was greatly induced and maintained a long period (1–7d) (Figure 7b).

### CO regulation of chlorotic phenotype depends on NO action

**Figure 8:**
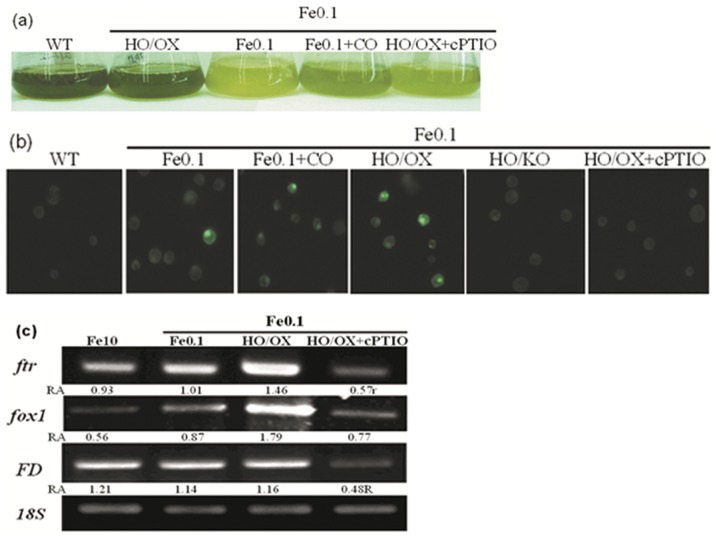
Carbon monoxide or heme oxygenase-regulated chlorophyll accumulation under Fe-deficiency was correlated with NO action. Cells were grown in the iron-sufficient Fe (10 µM) nutrient solution for 5 days, and then transferred to iron-limited Fe (0.1 µM) media with or without 8 µM CO for 8 days. Meanwhile, the CO-exposed cells were treated with 3 µM cPTIO. After that, (a): the cell were loaded with 15 µM 4,5-diaminofluo-rescence (DAF-2DA) for 15 min and immediately photographed (bar = 1 mm). (b): the cell phenotype was photographed. (c): the gene transcript levels. 18S was used for cDNA normalization. The number below the band indicates the relative abundance (RA) of the genes with respect to the loading control 18S.

Since NO has been reported to respond to iron-deficiency [15], it was of great interest to experimenting whether CO-regulating phenotype and iron homeostasis was associated with NO. Production of NO in cells was detected by using DAF-2DA fluorescent emission. Cells under iron-deficiency (Fe0.1) displayed light NO intensity in wild-type and HO/KO mutants, however, wild-type cells exposed to 8 µM CO (Fe0.1+CO) stained intense; moreover, HO/OX mutants showed strong fluorescence (Figure 8b). To ascertain whether CO-regulating phenotype was dependent on NO action, the NO specific scavenger cPTIO was used to treat *Chlamydomonas* cells in the presence of CO. It was shown cPTIO was able to prevent the CO-improving NO production and chlorophyll accumulation (Figure 8a), suggesting that NO might act downstream of CO. For further understanding, we tested the expression condition of *FTR1*, *FOX1*, and *FD*. The results displayed interestingly that their transcript levels were inhibited by cPTIO, which was consistent with the fluorescence variation tendency (Figure 8c).

## Discussion

The green alga *Chlamydomonas reinhardtii* is a photoautotrophic eukaryote, which is identical to higher plants in their many basic metabolisms [2]. It has been estimated that more than 90 percent of iron is located in the chloroplasts, because a high abundance of iron is required to maintain the structural and functional integrity of thylakoid membranes in chloroplasts [28]. Chlorophyll composes the major component of chloroplasts and is positively correlated with the iron status. Electron flow in thylakoids requires Fe-S clusters. Our results indicated that exogenous CO can promote the chlorophyll accumulation and greening in iron-deficient *Chlamydomonas*, which reflected the great improvement of iron status. The similar observation also can be found in *Chlamydomonas* transgenic mutants of HO/OX. These main consequences suggest that CO-improving iron homeostasis is a general response in green algae.

### CO or HO1 mediates intracellular iron availability

Previous studies indicated that iron deficiency caused the disruption of chloroplast ultrastructure and degradation of its protein components [29], [30]. Several steps in photosynthetic pigment synthesis and chloroplast ultrastructure establishment were dependent on iron availability [1]. In our study, *Chlamydomonas* cells under iron deficiency showed the chlorotic symptom, with a cell yellowing and a low level of chlorophyll content, which may represent the proteolytic loss of photosynthetic components [1]. Moreover, TEM micrographs from iron-deficient cells revealed chloroplasts with few photosynthetic lamellae and grana (Figure 2c), exhibiting a typical feature of thylakoid disruption caused by iron deficiency [29], [30]. Interestingly, most of normal stacks of thylakiods could be recovered by exposure to CO, even though the chloroplasts were under iron-deficiency. These results provided further evidence that CO was able to improve the pigment accumulation and prevent disruption of chloroplast structure through a putative mechanism in alga. An important role of iron in chloroplasts is associated with their thylakoid electron transfer chain. Several major metal-proteins containing heme or iron-sulphur clusters play crucial roles in supporting thylakoid electron transfer and chlorophyll biosynthesis. In animal cells, iron deficiency induced HO-1 expression [21]. Up-regulation of HO-1 expression under iron-deficiency suggests a catabolic process of heme with the concomitant recycling of iron in tissues, making more free iron available to the iron-starved cells. In this case, HO-1 might be a regulator for iron availability in Fe-stress cells. Our results with increased HO-1 expression under iron deficiency may help to understand that heme may be the source for iron mobilization within cells or tissues. Therefore, the HO1-mediated iron release from iron-pool is likely one of the important mechanisms for iron homeostasis. In addition to free Fe, carbon monoxide is another product of HO-1 and may affect Fe-containing proteins through a putative pathway. This speculation can be supported by our results that the HO/OX mutant cells could inhibit the chlorotic symptom and thylakoid disruption caused by iron deficiency completely when iron was limited (Figure 1b, 3b). In animals, iron-containing protein is the target of CO [17]. CO regulates Fe metabolism via its reactivity with iron-containing proteins with either heme or (Fe-S) clusters or through its ability to bind directly the metal ion [13], [21]. If an alga has a mechanism like that, HO1-derived CO may facilitate the delivery of iron from iron-containing components and recycling in cells.

### Iron accumulation and iron-related genes were up-regulated by CO or HO1

Iron is essential for green algae survival. Some iron-regulated proteins, such as ferric reductase and iron transporters play crucial roles in iron uptake and translocation in algae [2]. Our results showed that the activity of multi-copper ferroxidase 1 in *Chlamydomonas* cells was induced by CO exposure during the iron deficiency. The result was in agreement with the enhanced *FOX1* expression under the same condition. A recent study has shown that there was a positive correlation between *FOX1* expression and multi-copper ferroxidase activity in iron-deficient *Chlamodomonas* cells. Furthermore, Ftr1 (for iron transporter) and FD (necessary for electron distribution) were also found to be up-regulated by CO in the iron-starved cells, indicating that CO may mediate iron acquisition through the activation of the genes in iron-stressed plants. The mechanism for the CO-regulating gene expression is unknown, but the current results indicate that CO may affect gene expression through a putative pathway. Interestingly, CO-enhanced expression of *FOX1*, *FTR1* and *FD* could be abolished by NO scavenger cPTIO (Figure 8c). This result indicates that NO is possibly involved in the CO-mediating regulation of gene expression, strongly implying the cross-talk between CO and NO. It is possible that NO would be one of the components that act downstream of CO.

Owing to the fact that CO up-regulated the genes responsible for iron homeostasis, we assayed the iron accumulation in *C. reinharditii*. Cells treated with CO improved iron accumulation in the *C. reinharditii* cells and also the HO/OX mutants. This result is in agreement with increased amount of FOX1, FTR1 and FD transcripts. Notably, CO did not enhance iron accumulation in *Chlamydomonas* cells grown in sufficient-iron conditions (data not shown). Thus, CO or HO1 can confer iron homeostasis through either intracellular iron mobility or accumulation in algae.

### CO/HO1-improved iron homeostasis is dependent on NO action

Over the last few years, NO has been shown to play an important role in iron metabolism by its ability to increase IRP1-RNA binding activity and directly interact with the [4Fe–4S] cluster [13]. NO forms complexes with iron and removes iron from a range of Fe-containing proteins, including ferritin, ribonucleotide reductase or other heme-containing proteins [21]. In plants, several lines of evidence were shown to regulate the response of plants to iron starvation [14], [15]. In algae, NO was reported to regulate heavy metal stress [16]. Nevertheless, how iron circulates within algae in the presence of NO is largely unknown. Carbon monoxide can be endogenously generated in organisms at micromolar quantity [31]. Like NO, CO has a high affinity for iron and both share some chemical properties and indeed exert similar biological effects [13], [31]. Also, both CO and NO can bind to the heme centre of numerous Fe-containing proteins, such as the centre of soluble guanylate cyclase to increase their activity, and consequently regulate downstream events linked to iron metabolism [21]. With regard to the biochemical interaction between CO and NO, early report indicated that CO was able to stimulate NO release from proteins and produce peroxynitrite [32]. This implicates the mobility of intracellular NO at least from partial synergistic interactions of the two molecules [33], [34]. The current studies have demonstrated that CO-induced NO production in iron-deficient cells of *Chlamydomonas* and mutants, and the action could be prevented by NO scavenger cPTIO. Thus, our results suggested CO and NO may coordinately regulate iron-based metabolisms in some cases [32]–[36].

In general, we have demonstrated that carbon monoxide and HO1 was able to regulate iron homeostasis in alga under iron deficiency. Based on this observation, we speculated that CO, by its affinity for ferrous, can carry iron away from non-functional binding sites in the cells, making it physiologically available to the chloroplast. Moreover, CO-improved iron nutritional status is closely associated with NO action. CO may induce NO generation, and NO successively regulates Fe availability. Finally, CO may regulate the expression of genes that control iron acquisition and recycling. These results indicate CO regulates iron homeostasis most possibly through multiple pathways in *Chlamydomonas reinhardtii*.

## Materials and Methods

### Cell growth and treatment

*Chlamydomonas reinhardtii* (CC-503 cw92 mt+) were cultivated under the condition of 26±2°C, with a light intensity of 80 µmol m^−2^ s^−1^ and 14 h photoperiod [16]. All experiments were performed with exponentially growing culture. When treated, algae were incubated in the culture medium containing 10 µM Fe (sufficient) or 0.1 µM Fe (deficient) with or without CO for 8 days.

### Chlorophyll quantification

After treatment, liquid cells were collected (2 ml) and put into dark for 15 min. Then chlorophyll data were obtained by phytoplankton luminoscope (phyto-PAM).

### Transmission electron microscopy

Cells were fixed in 2.5% glutaraldehyde in 0.05 M potassium phosphate buffer (pH 7.1) for 8 h and post-fixed with OsO4. The samples were dehydrated in ethanol (95%, v/v) and embedded in Spurrs epoxy resin. Ultrathin sections were obtained using an ultramicrotome and stained with uranyl acetate and basic lead citrate for observation using a JEOL TEM [34].

### Iron quantification

Harvested cells (100 ml) were washed for 5 min in a solution containing 5 mM CaSO4 and 10 mM EDTA. Then digested completely in 70% HNO3 at 120°C. Iron was quantified by inductively coupled plasma spectrometry (Perkin Elmer Optimal 2100DV).

### Multi-copper ferroxidase 1 activity

Harvested cells were put into 10 µM cycloheximide for 2 h. After that, treated cells were transferred into Fe-sufficient or Fe-deficient mediam with or without 8 µM CO. pPD (phenylenediamine) is the substrate of Multi-copper ferroxidase 1 and the reaction started after adding pPD. The mixture was kept in dark and shaking for 3 h. After centrifuging, the enzyme activity was determined by measuring at 525 nm [35].

### Analysis of transcripts

Total RNA extraction and reverse transcription was referred to Kong's method [36]. Expression of 18S gene (accession number JF834705) was used as a control. RT-PCR conditions for 18S amplification were as follows: 94°C for 4 min, 94°C for 30 s, 58°C for 30 s, 72°C for 30 s, 72°C for 8 min, and 25 cycles. Each PCR experiment was performed three times with different cDNA sets from independent biological replicates. The PCR products were applied to 1% (w/v) agarose gel electrophoresis and stained with ethidium bromide. The primer pairs used for RT-PCR analysis of other genes were designed and the number of cycles was based on the follows: HO-1 (accession number XM_001702531), 30cycles; FOX1 (accession number AF450137), 25cycles; FTR1 (accession number AF478411), 27cycles; FD (accession number L10349), 25cycles.

### Detection of intracellular NO

Wild-type cells were exposed to CO then transferred to 20 mM Hepes-NaOH (pH 7.5) buffer solution containing 15 µm specific NO fluorescent probe 4, 5-diaminofluorescein diacetate (DAF-2DA), while and HO/OX mutant cells were transferred directly. After incubated in darkness for 15 min, the cells were washed several times and visualized immediately (excitation 490 nm and emission 525 nm) by a fluorescence microscope (Axio Imager. A1, Zeiss) [16], [30]–[36].

### Statistical analysis

Each result shown in figures was the mean of three replicated treatments, and each treatment contained at least 10^7^ cells ml^−1^. The significant differences between treatments were statistically evaluated by standard deviation and Student's t-test methods.

## References

[1] Briat JF, Curie C, Gaymard F (2007) Iron utilization and metabolism in plants. Curr Opin Plant Biol 10: 276–282. 17434791 10.1016/j.pbi.2007.04.003

[2] Merchant SS, Allen MD, Kropat J, Moseley JL, Long JC, et al (2006) Between a rock and a hard place: Trace element nutrition in Chlamydomonas. Acta Bioch Bioph 1763: 578–594. 10.1016/j.bbamcr.2006.04.007 16766055

[3] Eide D, Broderius M, Fett J, Guerinot ML (1996) A novel iron-regulated metal transporter from plants identified by functional expression in yeast. PNAS 93: 5624–5628. 8643627 10.1073/pnas.93.11.5624 PMC39298

[4] Robinson NJ, Procter CM, Connolly EL, Guerinot ML (1999) A ferric-chelate reductase for iron uptake from soils. Nature 397: 694–697. 10067892 10.1038/17800

[5] Henriques R, Jásik J, Klein M, Martinoia E, Feller U, et al (2002) Knock-out of Arabidopsis metal transporter gene IRT1 results in iron deficiency accompanied by cell differentiation defects. Plant Mol Biol 50: 587–597. 12374293 10.1023/a:1019942200164

[6] Xie YJ, Mao Y, Lai DW, Zhang W, Shen WB (2012) H_2_ Enhances Arabidopsis Salt Tolerance by Manipulating ZAT10/12-Mediated Antioxidant Defence and Controlling Sodium Exclusion. PLoS ONE 7 11: e49800. 23185443 10.1371/journal.pone.0049800 PMC3504229

[7] Lu Y, Liu TY, Li B, Sui Y, Chen JS, et al (2012) Comparative Sequence Analysis of the *Ghd7* Orthologous Regions Revealed Movement of *Ghd7* in the Grass Genomes. PLoS ONE 7 11: e50236. 23185584 10.1371/journal.pone.0050236 PMC3503983

[8] Connolly EL, Fett JP, Guerinot M (2002) Expression of the IRT1 metal transporter is controlled by metals at the levels of transcript and protein accumulation. Plant Cell 14: 1347–1357. 12084831 10.1105/tpc.001263 PMC150784

[9] Li L, Cheng X, Ling HQ (2004) Isolation and characterization of Fe (III)-chelate reductase gene LeFRO1 in tomato. Plant Mol Biol 54: 125–136. 15159639 10.1023/B:PLAN.0000028774.82782.16

[10] Wu H, Li L, Du J, Yuan Y, Cheng X, et al (2005) Molecular and biochemical characterization of the Fe (III) chelate reductase gene family in A*rabidopsis thaliana* . Plant Cell Physiol 46: 1505–1514. 16006655 10.1093/pcp/pci163

[11] Schmidt W (1999) Mechanisms and regulation of reduction-based iron uptake in plants. New Phytol 141: 1–26.

[12] Wang YH, Li XC, Ge QZ, Jiang X, Wang WD, et al (2012) Nitric Oxide Participates in Cold-InhibitedCamellia sinensis Pollen Germination and Tube Growth Partly via Cgmp *In Vitro* . PLoS ONE 7 12: e52436. 23272244 10.1371/journal.pone.0052436 PMC3525538

[13] Watts RN, Richardson DR (2004) Differential effects on cellular iron metabolism of the physiologically relevant diatomic effector molecules, NO and CO, that bind iron. Acta Bioch Bioph 1692: 1–15. 10.1016/j.bbamcr.2004.02.004 15158359

[14] Murgia I, Delledonne M, Soave C (2002) Nitric oxide mediates iron-induced ferritin accumulation in Arabidopsis. Plant J 30: 521–528. 12047627 10.1046/j.1365-313x.2002.01312.x

[15] Graziano M, Lamattina L (2007) Nitric oxide is required for molecular and physiological responses to iron deficiency in tomato roots. Plant J 52: 949–960. 17892445 10.1111/j.1365-313X.2007.03283.x

[16] Zhang LP, Mehta SK, Liu ZP, Yang ZM (2008) Copper-induced proline synthesis is associated with nitric oxide generation in *Chlamydomonas reinhardtii* . Plant Cell Physiol 49: 411–419. 18252734 10.1093/pcp/pcn017

[17] Boczkowski J, Poderoso JJ, Motterlini R (2006) CO–metal interaction: vital signaling from a lethal gas. Trends in Biochem Sci 31: 614–621. 16996273 10.1016/j.tibs.2006.09.001

[18] Siegel SM, Renwick G, Roson LA (1962) Formation of carbon monoxide during seed germinationand seedling growth. Science 137: 683–684. 17770955 10.1126/science.137.3531.683

[19] Guo K, Xia K, Yang ZM (2008) Regulation of tomato lateral root development by carbon monoxide and involvement in auxin and nitric oxide. J Exp Bot 59: 3443–3452. 18653694 10.1093/jxb/ern194 PMC2529230

[20] Davis SJ, Kurepa J, Vierstra RD (1999) The Arabidopsis thaliana HY1 locus, required for phytochrome-chromophore biosynthesis, encodes a protein related to heme oxygenases. PNAS 96: 6541–6546. 10339624 10.1073/pnas.96.11.6541 PMC26918

[21] Watts RN, Ponka P, Richardson DR (2003) Effects of nitrogen monoxide and carbon monoxide on molecular and cellular iron metabolism: mirror-image effector molecules that target iron. Biochem J 369: 429–440. 12423201 10.1042/BJ20021302 PMC1223127

[22] Muramoto T, Kohchi T, Yokota A, Hwang I, Goodman HM (1999) The Arabidopsis photomorphogenic mutant hy1 is deficient in phytochrome chromophore biosynthesis as a result of a mutation in a plastid heme oxygenase. Plant Cell 11: 335–347. 10072395 10.1105/tpc.11.3.335 PMC144190

[23] Merchant SS, Prochnik SE, Vallon O, Harris EH, Karpowicz SJ, et al (2007) The Chlamydomonas Genome Reveals the Evolution of Key Animal and Plant Functions. Science 318: 245–251. 17932292 10.1126/science.1143609 PMC2875087

[24] Allen MD, del Campo JA, Kropat J, Merchant SS (2007) FEA1, FEA2, and FRE1, encoding two homologous secreted proteins and a candidate ferrireductase, are expressed coordinately with FOX1 and FTR1 in iron-deficient *Chlamydomonas reinhardtii* . Eukaryot Cell 6: 1841–1852. 17660359 10.1128/EC.00205-07 PMC2043389

[25] Deng X, Eriksson M (2007) Two iron-responsive promoter elements control expression of FOX1 in *Chlamydomonas reinhardtii* . Eukaryot Cell 6: 2163–2167. 17905921 10.1128/EC.00324-07 PMC2168406

[26] La Fontaine S, Quinn JM, Nakamoto SS, Page MD, Göhre V, et al (2002) Copper-dependent iron assimilation pathway in the model photosynthetic eukaryote *Chlamydomonas reinhardtii* . Eukaryot Cell 1: 736–757. 12455693 10.1128/EC.1.5.736-757.2002 PMC126744

[27] Paz Y, Katz A, Pick U (2007) A multicopper ferroxidase involved in iron binding to transferrins in Dunaliella salina plasma membranes. J Biol Chem 282: 8658–8666. 17227764 10.1074/jbc.M609756200

[28] Kim SA, Guerinot ML (2007) Mining iron: Iron uptake and transport in plants. FEBS Lett 581: 2273–2280. 17485078 10.1016/j.febslet.2007.04.043

[29] Stocking CR (1975) Iron deficiency and the structure and physiology of maize chloroplasts. Plant Physiol 55: 626–631. 16659137 10.1104/pp.55.4.626 PMC541676

[30] Winder TL, Nishio JN (1995) Early iron deficiency stress response in leaves of sugar beet. Plant Physiol 108: 1487–1494. 7659749 10.1104/pp.108.4.1487 PMC157528

[31] Hartsfield CL (2002) Cross-talk between carbon monoxide and nitric oxide. Antioxid & Redox Sign 4: 301–307. 10.1089/152308602753666352 12006181

[32] Thom SR, Xu YA, Ischiropoulos H (1997) Vascular endothelial cells generate perxynitrite in response to carbon monoxide exposure. Chem Res Toxicol 10: 1023–1031. 9305585 10.1021/tx970041h

[33] Piantadosi CA (2002) Biological chemistry of carbon monoxide. Chem Res Toxicol 4: 259–270. 10.1089/152308602753666316 12006177

[34] Chen J, Shiyab S, Han FX, Monts DL, Waggoner CA, Yang ZM, Su Y (2009) Bioaccumulation and physiological effects of mercury in Pteris vittata and Nephrolepis exaltata. Ecotoxicology 18: 110–121. 18766440 10.1007/s10646-008-0264-3

[35] Ulrich E, Thomas JB (1998) Iron assimilation in Chlamydomonas reinhardtiiinvolves ferric reduction and is similar to Strategy I higher plants. J Exp Bot 49: 1219–1226.

[36] Kong WW, Zhang LP, Guo K, Liu ZP, Yang ZM (2010) Carbon monoxide improves adaptation of Arabidopsis to iron deficiency. Plant Biotechnol J 8: 88–99. 20055961 10.1111/j.1467-7652.2009.00469.x

